# Decision Fatigue in Dermatopathology: Cognitive Load and Diagnostic Vulnerability

**DOI:** 10.1111/cup.14866

**Published:** 2025-09-11

**Authors:** Cornelia Sigrid Lissi Müller

**Affiliations:** ^1^ MVZ for Histology, Cytology and Molecular Diagnostics Trier Trier Germany; ^2^ Faculty of Medicine Saarland University Homburg/Saar Germany

**Keywords:** cognitive load, decision fatigue, defensive medicine, dermatopathology, diagnostic accuracy

## 1 Theoretical Foundations of Decision Fatigue

Histopathological diagnosis is cognitively demanding, requiring complex pattern recognition, clinical integration, and decision‐making under time pressure. A largely overlooked factor in dermatopathology is *decision fatigue*—the decline in decision quality after sustained cognitive effort. Well described in psychology, it reflects mental exhaustion that impairs information processing, risk assessment, and judgment [1]. Various psychological models attempt to explain this phenomenon and describe the mechanisms behind it. *Decision fatigue* describes a cognitive state in which the quality of decisions decreases after prolonged stress. This phenomenon was first described by Baumeister and colleagues as part of the theory of ego depletion. They postulate that the ability to self‐regulate is a limited resource that can be depleted by prolonged cognitive effort. As a result of this depletion, individuals tend to make more impulsive decisions or avoid decisions altogether [2]. In clinical practice, for example, decision fatigue manifests itself in the tendency to prefer standard decisions or to postpone complex decisions. Studies show that medical staff under high decision‐making pressure tend to make defensive decisions or order additional, possibly unnecessary, diagnostic tests more frequently. This may be due to reduced cognitive capacity exhausted by sustained decision‐making demands. However, recent research suggests that the effects of decision fatigue are not exclusively due to the depletion of a limited self‐regulatory resource. Rather, factors such as motivation, individual beliefs about one's own willpower, and external environmental conditions also play a decisive role. For example, studies show that people who are convinced that their willpower is unlimited are significantly less susceptible to typical symptoms of decision fatigue [3, 4]. Another key model for explaining decision fatigue is the System 1/System 2 model developed by Daniel Kahneman [5]. Kahneman distinguishes between two types of thinking: System 1, which is fast, intuitive, automatic, and emotionally controlled. System 1 requires little cognitive effort. System 2 is characterized by slow, analytical, controlled, rational thinking and requires high cognitive resources. According to Kahneman, decision fatigue leads people to increasingly fall back on the fast, intuitive System 1 because the more strenuous System 2 is already exhausted or is avoided to save energy. As a result, decisions are less well thought out, more influenced by heuristics or routines, and potentially more prone to error [5]. Regardless of the exact cause, however, the observation remains consistent that long phases of intensive cognitive stress—as are typical in everyday medical diagnosis—can lead to a measurable deterioration in the quality of decision‐making [6]. These findings suggest a shift from a purely resource‐based model of decision fatigue to a more dynamic view that includes psychological factors. In dermatopathology, where many complex decisions are made under time pressure, this increases the risk of diagnostic errors. Understanding the underlying mechanisms is crucial for effective prevention and intervention.

## 2 Transfer to Dermatopathology

Dermatopathology is highly susceptible to decision fatigue due to high case volume, subtle findings, and limited clinical context. Complex entities like atypical melanocytic lesions or early lymphoproliferative disorders demand nuanced judgment, which is further challenged by cognitive fatigue. Dermatopathological diagnostics places particularly high cognitive demands as it operates at the interface between clinical dermatology and microscopic morphology. Numerous decision‐making situations in everyday dermatopathology are potentially susceptible to decision fatigue, especially when a large number of similar cases have to be processed without sufficient clinical contextual information [7]. A classic example is the assessment of melanocytic lesions. While the distinction between clearly benign nevi and clearly malignant melanomas is relatively clear in many cases, there is a broad diagnostic gray area spectrum ranging from “atypical nevi” to “melanocytic lesions of undetermined biological potential” [8]. These zones of uncertainty require a high level of concentration and differential diagnostic sensitivity. Under conditions of cognitive exhaustion, there is a risk of either overcalling or undercalling to shorten the decision‐making process. The diagnosis of inflammatory dermatoses is also characterized by a high degree of variability and is often confronted with unspecific patterns. Here, the risk of decision fatigue is particularly evident in the fact that findings are generally described as “non‐specific” or “compatible with dermatitis” without systematically documenting the differential diagnostic considerations that are necessary. The diagnostic depth typically decreases over the course of a long working day—an observation that has hardly been systematically investigated to date, but is repeatedly noticed in daily practice. There is also a tendency to order additional immunohistochemistry or additional molecular tests in difficult cases—in some cases less out of differential diagnostic necessity than as a psychological “relief mechanism” to cushion their own uncertainty. This form of defensive diagnostics can increase costs and processing times, on the one hand, and entail the risk of over‐interpretation of additional technical findings, on the other [9, 10]. Finally, decision fatigue is also reflected in the language used in diagnostic formulations. While differentiated assessments with clear recommendations for action dominate at the beginning of the working day, vague phrases such as *“*essentially unremarkable*”* or “to be correlated with a suitable clinic” are often used at the end of the day. Such formulations can be interpreted as a cognitive relief strategy, as they reduce the need for precise definitions. Studies from clinical medicine show that not only the decision‐making style but also the linguistic formulation changes with increasing mental fatigue [6, 11]. Although such data have not yet been obtained from dermatopathology or pathology, the findings suggest that linguistic clarity also decreases with increasing fatigue in histological diagnostics. Vague diagnoses may shift responsibility to clinicians without clear guidance, risking miscommunication and treatment delays. This highlights that decision fatigue in dermatopathology can impact diagnostic quality and clinical care, despite limited research. Structural factors in daily practice foster its unnoticed development. Dermatopathology laboratories often process hundreds of samples per day, from trivial excisions to shave biopsies to complex issues. Many of these cases are recurrently similar, leading to a monotonous cognitive load. Dermatopathology has been shown to exhibit high interobserver variability in challenging entities such as lichenoid dermatoses and atypical melanocytic lesions, often compounded by incomplete clinical information and high daily case volumes [12, 13, 14]. Moreover, Weyers critically highlights that the ambition for precise histopathological classification often borders on illusion, underscoring the limitations and subjectivity inherent in our field [15]. The quantitative workload forces dermatopathologists to make decisions at high frequency and often under time pressure. At the same time, dermatopathology is characterized by extreme morphological variability. Many inflammatory and neoplastic dermatoses are histologically similar or overlapping, while at the same time, additional clinical information such as exact localization, course, previous diagnoses, or treatment details are often incomplete or not available at all. The need to decide “in a vacuum” further increases the cognitive load, as dermatopathologists must constantly make implicit assumptions about the clinical context—a typical breeding ground for heuristics and poor decisions, especially in phases of exhaustion. Unlike in many technical disciplines, dermatopathological diagnosis remains subjective. Numerous studies show interobserver and intraobserver variability, even among experienced experts [16, 17]. This fundamental level of variability is further increased by decision fatigue, as individual fluctuations in judgment are exacerbated by fatigue [7, 10, 18]. In dermatopathology, missing feedback loops hinder learning and self‐assessment: errors often go unnoticed, positive feedback is rare, and judgment remains uncalibrated. Combined with high workload, uncertainty, and limited clinical context, this fosters decision fatigue.

## 3 Strategies to Mitigate Decision Fatigue: Lessons From Cognitive Science

Several evidence‐based strategies can mitigate decision fatigue: scheduling complex decisions early in the day, taking brief cognitive breaks, grouping tasks by complexity, minimizing multitasking and distractions, and fostering metacognitive self‐monitoring and peer consultation. While not yet specifically studied in dermatopathology, these approaches offer a transferable framework for optimizing diagnostic decision‐making [19, 20, 21].

## 4 Conclusion

Cognitive load is a subtle yet powerful factor in dermatopathology, influencing decisions, leading to vague wording, overcaution, or overinterpretation—especially under pressure, fatigue, or limited information. This is not individual failure, but a systemic issue that remains underrecognized. While resilience varies among diagnosticians, the phenomenon of decision fatigue is plausible and transferable from clinical medicine to dermatopathology. Pathology reports are not absolute truths but products of complex cognitive processes shaped by specific working conditions. Clinical input—history, comorbidities, prior findings, and images—is essential for context‐based diagnostics. Sharing diagnostic uncertainty is not weakness, but professional responsibility.

To counter decision fatigue, greater awareness of cognitive limits, structured breaks, and transparent communication are first steps. Interdisciplinary training and feedback systems can further support diagnostic precision and reduce overload, particularly in high‐throughput environments.

## Ethics Statement

The author has nothing to report.

## Conflicts of Interest

The author declares no conflicts of interest.

## Data Availability

Data sharing not applicable to this article as no datasets were generated or analysed during the current study.
